# RT-qPCR-based pool testing for the diagnosis of COVID-19

**DOI:** 10.31744/einstein_journal/2023AE0115

**Published:** 2023-06-23

**Authors:** Hugo Itaru Sato, Murilo Soares Costa, Ricardo Hiroshi Caldeira Takahashi, Karine Lima Lourenço, Nathalia Sernizon Guimarães, Claudia Regina Lindgren Alves, Elaine Leandro Machado, Unaí Tupinambás, Flávio Guimarães da Fonseca, Santuza Maria Ribeiro Teixeira

**Affiliations:** 1 Vaccine Technology Center Universidade Federal de Minas Gerais Belo Horizonte MG Brazil Vaccine Technology Center, Universidade Federal de Minas Gerais, Belo Horizonte, MG, Brazil.; 2 Postgraduate Program in Infectious Diseases and Tropical Medicine Faculdade de Medicina Universidade Federal de Minas Gerais Belo Horizonte MG Brazil Postgraduate Program in Infectious Diseases and Tropical Medicine, Faculdade de Medicina, Universidade Federal de Minas Gerais, Belo Horizonte, MG, Brazil.; 3 Mathematics Department Instituto de Ciências Exatas Universidade Federal de Minas Gerais Belo Horizonte MG Brazil Mathematics Department, Instituto de Ciências Exatas, Universidade Federal de Minas Gerais, Belo Horizonte, MG, Brazil.; 4 Graduate Program in Collective Health Universidade Federal da Bahia Salvador BA Brazil Graduate Program in Collective Health, Universidade Federal da Bahia, Salvador, BA, Brazil.; 5 Department of Pediatrics Faculdade de Medicina Universidade Federal de Minas Gerais Belo Horizonte MG Brazil Department of Pediatrics, Faculdade de Medicina, Universidade Federal de Minas Gerais, Belo Horizonte, MG, Brazil.; 6 Department of Preventive and Social Medicine Faculdade de Medicina Universidade Federal de Minas Gerais Belo Horizonte MG Brazil Department of Preventive and Social Medicine, Faculdade de Medicina, Universidade Federal de Minas Gerais, Belo Horizonte, MG, Brazil.; 7 Medical Clinic Department Faculdade de Medicina Universidade Federal de Minas Gerais Belo Horizonte MG Brazil Medical Clinic Department, Faculdade de Medicina, Universidade Federal de Minas Gerais, Belo Horizonte, MG, Brazil.

**Keywords:** COVID-19, Coronavirus infections, SARS-CoV-2, Pandemics, Reverse transcriptase polymerase chain reaction

## Abstract

This study proposes a strategy for large-scale testing among a large number of people for the early diagnosis of COVID-19 to elucidate the epidemiological situation. Pool testing involves the analysis of pooled samples. This study aimed to discuss a reverse transcription technique followed by quantitative real-time polymerase chain reaction using pool testing to detect SARS-CoV-2 in nasopharyngeal swab samples. The study proposes an innovative diagnostic strategy that contributes to resource optimization, cost reduction, and improved agility of feedback from results.

## Highlights

Pool testing is simultaneously performed on multiple samples to efficiently and cost-effectively detect COVID-19. Pool testing can optimize resource utilization and expand diagnostic access, and is a viable alternative for developing countries with limited access to testing. To optimize resources, the pool size was determined by estimating COVID-19 prevalence in the study population.

**Figure:**
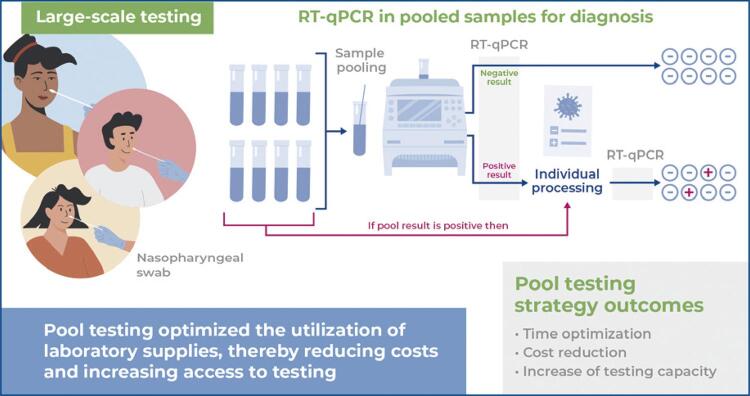

## INTRODUCTION

The reverse transcription technique followed by quantitative real-time polymerase chain reaction (RT-qPCR), indicated to be performed within three to five days of the onset of symptoms or suspicion of contagion, is the main confirmatory test for diagnosing COVID-19, which is caused by severe acute respiratory syndrome coronavirus 2 (SARS-CoV-2).^(1,2)^ However, the use of this test in low- and middle-income countries has several limitations, such as technical complexity owing to the need for infrastructure with an adequate level of biosafety, and the high cost of equipment and supplies. These limitations complicate access to the timely diagnosis of symptomatic and asymptomatic cases, which is a major challenge in managing the pandemic.

Pool testing is a strategy used to group samples for laboratory testing. Individual samples were collected and then pooled in the laboratory for processing and analysis. If the RT-qPCR test results of the sample pool were positive, individual samples within the pool were analyzed to identify those that had detectable viral RNA levels. If the results were negative, all the samples were considered to have undetectable viral RNA levels.^(1)^ The pool testing strategy was developed by Dorfman to detect syphilis in soldiers during World War II.^(3)^ This strategy can facilitate population-wide testing, thereby optimizing resources and reducing laboratory costs. This would, in turn, elucidate the epidemiological situation and provide a reference for measures aimed at reducing the circulation and transmission of infectious agents and controlling the emergence of outbreaks, thus contributing to the fight against epidemics.^(1)^

Studies show that large-scale continuous testing aimed at the early diagnosis of COVID-19 is a primary strategy in epidemiological surveillance, contributing decisively to assessing the evolution of the pandemic, incidence rate, mortality, transmissibility, lethality, and identification of suspected cases.^(4)^ Considering the complexity of controlling the COVID-19 pandemic and the high cost of performing RT-qPCR tests in Brazil, this study aimed to discuss the performance of RT-qPCR in pool testing performed at (CT Vacinas/UFMG - http://www.ctvacinas.ufmg.br/) in partnership with the UPA Centro-Sul from Belo Horizonte, Minas Gerais for the detection of SARS-CoV-2. The successful implementation of this method serves as a model for many other pathologies and epidemics.

## POOL TESTING

Trained professionals collected nasopharyngeal swabs from suspected cases of SARS-CoV-2 infection. A swab from each patient was then immersed in a tube containing 1mL of viral inactivation and transport solution^(5)^ and the same was processed and analyzed in the laboratory within 18 hours.

Within a laboratory structure with an adequate biosafety level, a previously trained technician prepared the pools to perform RT-qPCR, which involved adding 50µL of each sample to 1.5mL microtubes. The remaining samples were stored for further analysis, depending on the results of the pooled test. The number of samples per pool was determined by estimating the prevalence of COVID-19 in the study population, which ranged from 3 to 16 samples per pool. After homogenization, RNA extraction and RT-qPCR were performed as they would have been on a single sample, considering the maximum sample volume for processing using an RNA extraction column to be 140µL.

For RNA extraction and purification, the protocol outlined in the “QIAamp Viral RNA Mini Kit” (QIAGEN, Germany) was used. After cDNA synthesis, using a QuantStudio 5 real-time thermocycler PCRs were performed with two targets, the endogenous human RNase P gene and the E gene, which encodes the viral envelope, following the protocol outlined by Charité.^(6)^

**Figure 1:**
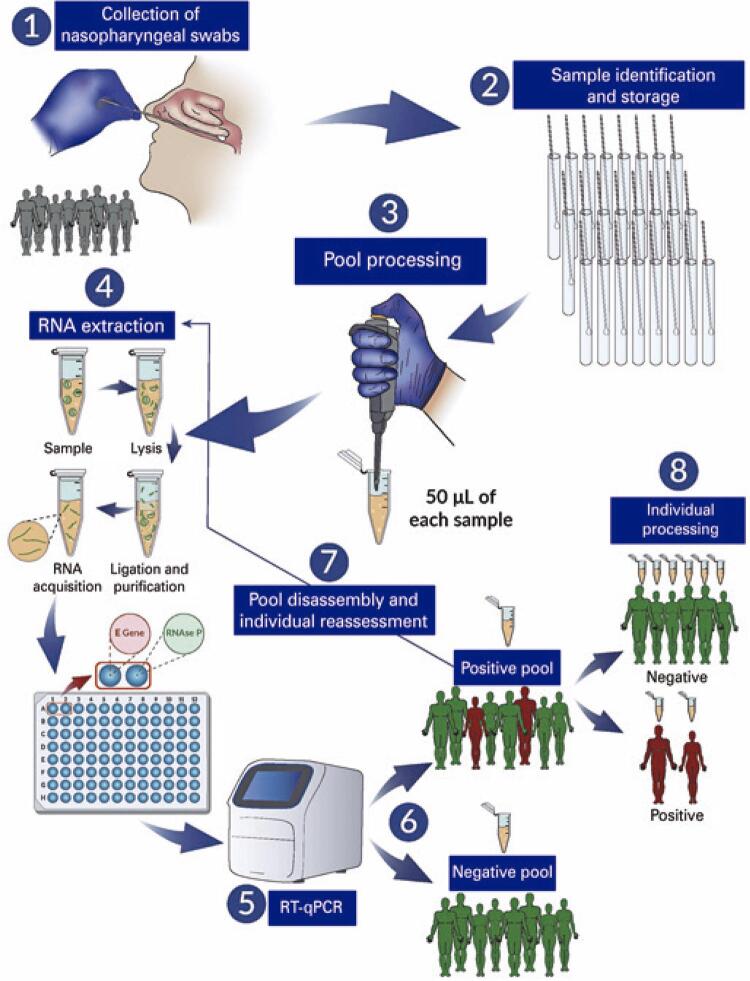
Strategy for performing reverse transcription and quantitative real-time polymerase chain reaction in pooled testing

In cases where the pool testing result yielded detectable viral RNA levels, samples in the group were individually processed using 140µL of each sample to extract the RNA. When the pool testing result yielded undetectable viral RNA levels, all samples in the pool were assigned the same results, thus not necessitating individual sample testing as shown in figure 1. Pool tests with inconclusive results owing to the absence of endogenous gene amplification necessitate pool reassembly. Pool testing results that presented a small amount of initial viral RNA, with Cq values close to the detection threshold of the method (the detection limit or cut-off point may vary according to the kits used in each laboratory, owing to differences such as enzymatic efficiency, yield, and quality of the extracted RNA), samples in those given pool were processed individually. Test cut-off points were set as described by Costa et al.^(7)^ After result analysis, the samples were stored at -80^o^C in an inactivation transport medium. The purified RNA samples were also stored at -80^o^C.

The results were obtained and released within 72 hours of arrival at the laboratory. Figure 1 presents the RT-qPCR in pooled testing strategy.

## DISCUSSION

Timely diagnosis of COVID-19 is essential to managing the pandemic and is mandatory in every suspected case, despite the increased vaccination of the global population. Early diagnosis is essential to epidemiological surveillance and curbing the spread of the virus by adopting social distancing and isolation strategies after a detectable result.^(2)^

Mass testing through pool testing can be adopted as a public health measure with the help of central, regional, municipal, or supplementary health laboratories. This suggestion is justified by the consequent optimization of resources and inputs when pool testing results yield undetectable viral RNA levels (Step 3 in Figure 1), based on the size of the pool and proceeding with the RT-qPCR technique as if it were a single sample (Steps 4 and 5 in Figure 1).

For sample pooling, the prevalence of the condition being investigated is estimated based on predictive mathematical models. The number of samples per group is adjusted to increase the identification of undetectable pools (Step 6 in Figure 1). This is because in populations where the prevalence of the investigated condition is low, a larger number of samples can be pooled, and there is a greater probability of the test results yielding undetectable viral RNA levels while maintaining sensitivity to detect the viral RNA as observed in the characterization of sample pools and individual sample testing in previous studies,^(7)^ thereby reducing costs, which can be estimated in different scenarios based on the number of positive pools,^(8)^ optimizing resources and expanding access to the diagnostic test.^(1)^

Based on its application in Brazil and developing countries, the pool testing strategy is a viable alternative that must be widely disseminated, as reagents for performing RT-qPCR are not locally produced and must be imported, which explains the high cost per test. In addition, there has been a shortage in the supply of these inputs at various times during the pandemic.

## CONCLUSION

Pool testing optimized the utilization of laboratory supplies, thereby reducing costs and increasing access to testing. This strategy can be used in the return of face-to-face teaching in addition to periodic surveillance for health professionals and other relatively vulnerable scenarios, such as long-stay institutions for older adults and other marginalized populations. As the COVID-19 pandemic is one of the greatest public health challenges ever faced, all effective and evidence-based measures must be analyzed to support health surveillance actions for the detection and control of the disease. Finally, the use of this strategy can be extended to the diagnosis of other diseases, particularly in epidemics where the demand for testing is high.
